# Intrinsicoid Deflection of the QRS Complex Predicts Appropriate Antitachycardia Pacing or Shock Therapy in Heart Failure Patients with an Implantable Cardioverter Defibrillator

**DOI:** 10.21470/1678-9741-2025-0081

**Published:** 2026-02-13

**Authors:** Meltem Altınsoy, İsmail Adsız, Hamza Sunman, Çagatay Tunca, Funda Basyigit

**Affiliations:** 1 Ankara Etlik City Hospital, Etlik, Ankara, Turkey

**Keywords:** Stroke Volume, Left Ventricular Function, ROC Curve, Implantable Defibrillators, Electrocardiography, Cardiac Sudden Death, Adenosine Triphosphate.

## Abstract

**Introduction:**

Sudden cardiac death remains a significant risk for patients with heart failure (HF). Current guidelines recommend implantable cardioverter defibrillator (ICD) for patients with low left ventricular ejection fraction (LVEF). However, the effectiveness and necessity of ICDs in patients with normal LVEF raise questions, especially given associated complications and costs.

**Objective:**

This study aims to evaluate the electrocardiographic predictors of appropriate ICD therapy (antiTachycardia pacing [ATP]/shocks) in patients with HF.

**Methods:**

We conducted an analysis of 160 consecutive HF patients (New York Heart Association class I-III, LVEF ≤ 35%) undergoing ICD controls from January 2023 to December 2024. Patients were classified into two groups based on the occurrence of appropriate ATP or ICD shocks. Electrocardiographic parameters including QRS duration, QTc interval, intrinsicoid deflection (ID), and fragmented QRS (fQRS) were assessed. Statistical analyses, including receiver operating characteristic curves and logistic regression, were performed to identify independent predictors of appropriate ICD therapy.

**Results:**

The ATP/shock group exhibited significantly prolonged QRS and QTc intervals, increased ID, and higher Selvester scores compared to the non-shock group. Notably, an ID > 50 ms emerged as a strong predictor of ICD therapy (sensitivity 96.3%, specificity 95.3%). Univariate and multivariate analyses identified ID, fQRS, and Selvester score as independent predictors of appropriate ICD therapy.

**Conclusion:**

Elevated ID, alongside other electrocardiographic parameters, serves as a valuable predictor for appropriate ICD therapy in HF patients. These findings support the potential for refining ICD implantation criteria, emphasizing the importance of detailed electrocardiographic evaluation in predicting arrhythmic events.

## INTRODUCTION

**Table:** 

Abbreviations, Acronyms & Symbols				
ALT	= Alanine aminotransferase		ICD	= Implantable cardioverter defibrillator
AST	= Aspartate aminotransferase		ID	= Intrinsicoid deflection
ATP	= AntiTachycardia pacing		LAD	= Left atrial diameter
AUC	= Area under the ROC curve		LV	= Left ventricular
BBB	= Bundle branch block		LVD	= Left ventricular diameter
BNP	= Brain natriuretic peptide		LVDD	= Left ventricular diastolic diameter
CAD	= Coronary artery disease		LVEF	= Left ventricular ejection fraction
CI	= Confidence interval		LVH	= Left ventricular hypertrophy
CRP	= C-reactive protein		MRA	= Mineralocorticoid receptor antagonist
CVD	= Cerebrovascular disease		NYHA	= New York Heart Association
DID	= Delayed intrinsicoid deflection		RDW-SD	= Red cell distribution-standard deviation
DM	= Diabetes mellitus		ROC	= Receiver operating characteristic
ECG	= Electrocardiogram		SCA	= Sudden cardiac arrest
FLN	= Fragmented lead number		SCD	= Sudden cardiac death
fQRS	= Fragmented QRS		SS	= Selvester score
Hb	= Hemoglobin		Tp-e	= T peak-T end
HF	= Heart failure		VF	= Ventricular fibrillation
HT	= Hypertension		VT	= Ventricular tachycardia

Sudden cardiac death (SCD) continues to be a significant cause of mortality in individuals with heart failure (HF)^[1]^. Implantable cardioverter defibrillator (ICD) implantation is recommended for patients who have received a minimum of three months of guideline-directed optimal medical therapy and continue to exhibit symptoms classified as New York Heart Association (NYHA) class II-III, with a left ventricular ejection fraction (LVEF) of ≤ 35%, irrespective of whether the underlying cause is ischemic or nonischemic cardiomyopathy, to avert SCD. Recommendations also advocate for the utilization of a preventive ICD in individuals with ischemic heart illness categorized as NYHA class I and having an LVEF ≤ 30%^[1,2]^. However, there is ongoing debate regarding the implantation of an ICD to prevent SCD in patients with low LVEF, as many victims of SCD have higher LVEF than guideline recommended LVEF cutoff^[3]^. Moreover, patients experience many complications (endocarditis, lead extraction, and revision) due to ICD implantation without having any survival benefit by adding financial cost to the health insurance system^[4]^. As for solution, some scoring systems are developed to predict SCD and identify HF subgroups that would be most beneficial from prophylactic ICD before ICD implantation, such as MADIT-II Trial-Based Risk Stratification Score (or MRSS), MADIT-ICD Benefit Score, Seattle Heart Failure Risk Model (or SHFM) and Seattle Proportional Risk Model (or SPRM), Heart Failure Meta-Score, SHOCKED Score, PACE, and Charlson Comorbidity Index-Based Scores^[5-7]^.Currently, there is no guideline-recommended scoring system for ICD implantation in patients with HF to prevent SCD.

Previous studies have shown that a delayed intrinsicoid deflection (DID) of ≥ 0.05 s in the lateral precordial leads V5 and V6 is associated with left ventricular hypertrophy (LVH), and it is included in the Romhilt-Estes criteria for the electrocardiographic diagnosis of LVH^[8]^. Voltage-dependent LVH and anatomical or mass-dependent LVH may exhibit unique and intersecting influences on the incidence of atrial and ventricular arrhythmias. Recently, Darouian et al.^[9]^ reported that DID was associated with increased sudden cardiac arrest (SCA) risk independent of echocardiographic LVH, electrocardiographic LVH, and reduced LVEF. Some electrocardiographic markers have emerged to predict SCA such as an increased resting heart rate and prolongation of the QRS, QTc, and JTc intervals. Intrinsicoid deflection (ID), or R-wave peak time, denotes the early phase of ventricular depolarization. The interval is defined as the time from the onset of the QRS complex to the apex of the R wave.

The aim of this study is to investigate electrocardiographic parameters, specifically ID, as predictors of appropriate ICD therapy in patients with ischemic and non-ischemic cardiomyopathy. We seek to determine the relationships between these parameters and the likelihood of receiving appropriate ICD therapy (antiTachycardia pacing [ATP]/shock), to identify cutoff values for key metrics, and to elucidate the underlying mechanisms contributing to arrhythmic risk in this patient population.

## METHODS

Our study involved 160 consecutive patients who sought ICD controls between January 2023 and December 2024. The study population was categorized into two groups depending on the presence of ATP or appropriate ICD shocks. Patients who had ATP or appropriate ICD shock due to ventricular tachycardia (VT)/ventricular fibrillation (VF) were the case group. Patients who have never received an ATP or ICD shock at least five years from the first implantation encompassed the control group. All patients had primary prevention indications according to the European Society of Cardiology Guidelines on cardiac pacing and cardiac resynchronization therapy for ICD at the time of implantation^[10]^. The study incorporated patients aged over 18 years who had either ischemic or non-ischemic cardiomyopathy and chronic stable HF classified as NYHA class I-III, with LVEF ≤ 35%. Patients who were receiving ICD treatment for secondary prophylaxis, as well as those with hypertrophic cardiomyopathy, congenital heart disease, or arrhythmic syndromes, were excluded from the study. Informed consent was obtained from the patients prior to their inclusion in the study.

The study adhered to the principles established in the Declaration of Helsinki and received approval from the Institutional Ethics Committee, reference number 2024-973.

### Electrocardiography

All the patients had standard resting 12-lead electrocardiograms (ECGs) (25 mm/s, 10 mm/mV). ECGs were evaluated by two general cardiologists who were unaware of the patient's medical background. We utilized Cardiocaliper version 3 to ensure an exact determination of ECG parameters. Results were compared at the end of the ECG analysis, and in case of disagreement, a third cardiologist's opinion was taken.

**Fig. 1:**
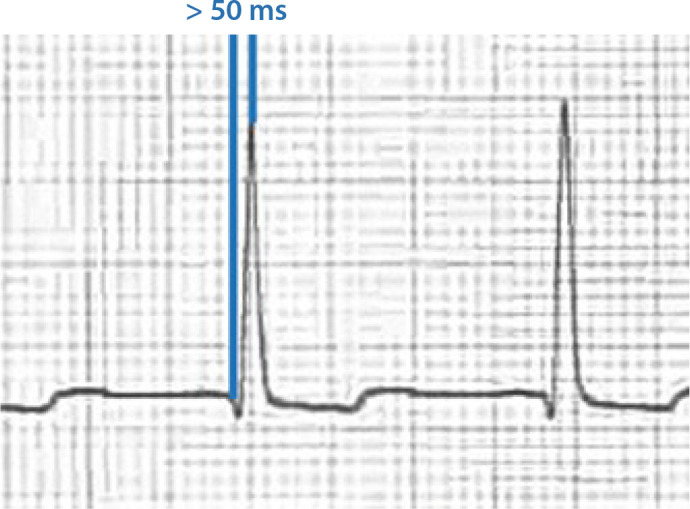
Measurement of intrinsicoid deflection.

ID duration is measured V5 or V6 from the onset of QRS depolarization until the first change of polarity (with both positive or negative QRS deflection) (Figure 1). It represents the time taken for excitation to spread from the endocardial to the epicardial surface of the left ventricle. Fragmentation of narrow QRS (< 120 ms) is characterized by the presence of an extra R wave (R') or notching at the nadir of the S wave or the occurrence of several R' waves in two adjacent leads that correspond to a major coronary artery area on the resting 12-lead ECG. Fragmentation of wide complex QRS (> 120 ms) encompasses many RSR patterns, characterized by the presence of more than two R waves (R") or exceeding two notches in the R wave, as well as more than two notches in either the downstroke or upstroke of the S wave^[11]^.

The revised Selvester QRS scoring system was created to assess infarct size utilizing 37 ECG variables to derive a cumulative score ranging from 0 to 29. We implemented the conditions as previously specified^[12]^. LVH was analyzed from the ECG via Sokolow-Lyon criteria (SV1 +RV5 or RV6 ≥ 35 mm)^[13]^.The frontal QRS-T angle, which is computed by measuring the absolute value of the difference between the QRS axis and the T-wave axis on a 12-lead ECG manually, represents the difference in orientation between ventricular depolarization and repolarization^[14]^. The T peak-T end (Tp-e) interval represents the transmural dispersion of repolarization.

### Echocardiography

Echocardiographic imaging was conducted in the left lateral decubitus position utilizing parasternal and apical views with a commercially available device (Vivid 7, GE Medical System, Horten, Norway; 3.5-MHz phased array transducer). LVEF was obtained from apical 4-chamber, 2-chamber, and parasternal long axis view. Echocardiographic LVH was calculated from the formula (left ventricular [LV] mass = 0.8(1.04([LV internal diameter in diastole + posterior wall thickness in diastole + interventricular septal thickness in diastole]3 - [LV internal diameter in diastole]3)) + 0.6 grams). Cutoffs are 134 g/m^2^ for men and 110 g/m^2^ for women^[15]^.

### ICD Interrogation

The end points for the study were appropriate ICD ATP/shocks due to ventricular tachyarrhythmias. All recorded occurrences of arrhythmia occurring since the device's implantation were retrospectively examined for detection accuracy, diagnostic precision, and the appropriateness of device therapy. The ICD programming included therapy for standard VT with ATP (three times Bursts, eight pulses at 85% VT cycle length) combined with low-energy shock therapy and for VF shock therapy with a 300-ms cutoff cycle interval. It was considered standard VT in the presence of sustained tachycardia with a cycle interval ranging from 300 to 400 ms, not discriminated as supra-VT by specific algorithms. It was considered VF when the cycle interval was < 300 ms. Appropriate ICD shock was defined as therapy for rapid sustained VT or VF episode.

### Statistical Analysis

Continuous variables are introduced as mean ± standard deviation or median with interquartile range, while categorical variables are expressed as percentages. The Kolmogorov-Smirnov test was utilized to assess the normal distribution of continuous variables. Categorical variables were analyzed using the chi-square test. Comparisons of continuous variables between the two groups were performed using the Student's *t*-test for parametric data and the Mann-Whitney U test for non-parametric data. The area under the receiver operating characteristic curve (ROC) analysis and comparison of ROC curves were performed using the MedCalc program (MedCalc Software Ltd). The DeLong test was used to compare the area under the ROC curve (AUC). Univariate and multivariate regression analyses were conducted to assess the relationship between clinical, electrocardiographic, and echocardiographic variables and the occurrence of ICD shocks. Statistical analyses were executed using IBM SPSS Statistics for Windows, version 22.0 (IBM Corp., Armonk, N.Y., USA). A *P*-value < 0.05 was considered statistically significant.

## RESULTS

We analyzed 160 consecutive HF patients with ICD and separated them into ATP/shock (+) group (male 81.5%, median age 66.0 ± 19.0 years) and ATP/shock (-) group (male 75.5%, median age 65.0 ± 24.0 years).

Table 1 summarizes their demographic, clinical, and laboratory values. There was no significant difference between the two groups in terms of age, sex, and comorbidities. Aspartate aminotransferase (21.5 ± 12.0 *vs.* 20.0 ± 10.0, *P* = 0.012), hs-troponin (56.0 ± 132.7 *vs.* 20.5 ± 32.5, *P* < 0.001), and calcium (8.88 ± 0.61 *vs.* 8.63 ± 0.56, *P* = 0.011) were higher in the ATP/shock (+) group than in the ATP/shock (-) group. The ATP/shock (-) group was more on mineralocorticoid receptor antagonist (MRA) and statin therapy (88.7% *vs.* 75.9%, *P* = 0.036; 95.3% *vs.* 81.5%, *P* = 0.005).

**Table 1:** Baseline characteristics of patients with ICD.

Characteristics	ICD Shock (+)	ICD Shock (-)	*P*-value
	n = 54	n = 106	
Age, years	66.0 ± 19.0	65.0 ± 13.0	0.555
Sex, male, n (%)	44 (81.5)	80 (75.5)	0.389
HT, n (%)	43 (79.6)	73 (68.9)	0.149
DM, n (%)	16 (29.6)	38 (35.8)	0.549
CAD, n (%)	47 (87.0)	86 (81.1)	0.346
CVD, n (%)	5 (9.3)	5 (4.7)	0.262
Glucose, mg/dl	116.0 ± 58.0	110.5 ± 52	0.528
Creatinine, mg/dl	0.95 ± 0.60	1.00 ± 0.40	0.865
Sodium, mmol/l	138.5 ± 3.0	139.0 ± 4.0	0.493
Potassium, mmol/l	4.3 ± 0.7	4.4 ±0.7	0.153
Calcium, mg/dl	8.88 ± 0.61	8.63 ± 0.56	0.011
Magnesium, mol/l	1.94 ± 0.30	1.90 ± 0.30	0.105
AST, u/l	21.5 ± 12.0	20.0 ± 10.0	0.012
ALT, u/l	21.0 ± 17.0	17.0 ± 11.0	0.240
Albumin, g/dL	40.0 ± 6.9	41.0 ± 4.5	0.468
Hb, g/dl	13.8 ± 2.8	13.4 ± 3.1	0.198
Platelet, 10^3/ul	216.5 ± 113.3	212.0 ± 68.3	0.816
Neutrophil, 10^3/ul	7.09 ± 4.13	5.80 ± 2.55	0.202
Lymphocyte, 10^3/ul	1.87 ± 1.31	1.80 ± 1.00	0.934
Monocyte, 10^3/ul	0.72 ± 0.42	0.71 ± 0.30	0.460
RDW-SD, fL	46.6 ± 8.0	46.5 ± 8.0	0.665
hs-troponin T, ng/l	56.0 ± 132.7	20.5 ± 32.5	< 0.001
NT-ProBNP, pg/ml	1300 ± 3023	858 ± 3170	0.394
CRP, mg/l	13.0 ± 23.8	7.8 ± 14.7	0.146
Drugs, n (%)			
RAS blocker	52 (96.3)	100 (94.3)	0.591
Beta blocker	52 (96.3)	102 (96.2)	0.982
Statin	44 (81.5)	101 (95.3)	0.005
Antiaggregant	42 (77.8)	91 (85.8)	0.197
Diuretic	38 (70.4)	83 (78.3)	0.269
Amiodarone	8 (14.8)	6 (5.7)	0.053
Ivabradine	5 (9.3)	8 (7.5)	0.708
MRA	41 (75.9)	94 (88.7)	0.036

ALT=alanine aminotransferase; AST=aspartate aminotransferase; BNP=brain natriuretic peptide; CAD=coronary artery disease; CRP=C-reactive protein; CVD=cerebrovascular disease; DM=diabetes mellitus; Hb=hemoglobin; HT=hypertension; ICD=implantable cardioverter defibrillator; MRA=mineralocorticoid receptor antagonist; RDW-SD=red cell distribution-standard deviation

QRS interval (127.0 ± 39.0 *vs.* 111.0 ± 34.0, *P* = 0.004), QTc interval (454.0 ± 43.0 *vs.* 433.0 ± 40.0, *P* < 0.001), Tp-e interval (90.0 ± 37.0 *vs.* 80.0 ± 37.0, *P* = 0.014), fragmented lead number (FLN) (3.5 ± 3.0 *vs.* 0.0 ± 1.0, *P* < 0.001), ID (60.0 ± 15.0 *vs.* 23.0 ± 19.0, *P* < 0.001), Selvester score (7.0 ± 4.0 *vs.* 4.0 ± 5.0, *P* < 0.001), and left ventricular diastolic diameter (LVDD) (60.5 ± 13.0 *vs.* 56.0 ± 8.0, *P* = 0.001) were higher in the ATP/shock (+) group than in the ATP/shock (-) group (Table 2).

**Table 2:** Electrocardiographic and transthoracic echocardiographic data of patients with ICD.

	ICD Shock (+)	ICD Shock (-)	*P*-value
	n = 54	n = 106	
Electrocardiography			
QRS interval,	127.0 ± 39.0	111.0 ± 34.0	0.004
QTc interval	454.0 ± 43.0	433.0 ± 40.0	< 0.001
Tp-e interval	90.0 ± 37.0	80.0 ± 37.0	0.014
BBB, n (%)	14 (25.9)	23 (21.7)	0.549
Frontal QRS-T angle	104.5 ± 112.0	88.0 ± 106.0	0.420
FLN	3.5 ± 3.0	0.0 ± 1.0	< 0.001
Intrinsicoid deflection	60.0 ± 15.0	23.0 ± 19.0	< 0.001
Sokolow-Lyon Score	1.01 ± 1.13	1.46 ± 1.38	0.007
Selvester Score	7.0 ± 4.0	4.0 ± 5.0	< 0.001
Echocardiography			
Diastolic diameter	60.5 ± 13.0	56.0 ± 8.0	0.001
LVEF, %	30.0 ± 13.0	30.0 ± 8.0	0.864
LAD, mm	44.0 ± 8.0	42.0 ± 9.0	0.066
LVH, n (%)	24 (44.4)	36 (34.0)	0.195

BBB=bundle branch block; FLN=fragmented lead number; ICD=implantable cardioverter defibrillator; LAD=left atrial diameter; LVEF=left ventricular ejection fraction; LVH=left ventricular hypertrophy; Tp-e=T peak-T end

The cutoff value of ID for ICD ATP/shock therapy was 50 msec. ID > 50 msec predicted ICD ATP/shock therapy with a sensitivity of 96.3% and specificity of 95.3% (*P* < 0.001).

**Fig. 2:**
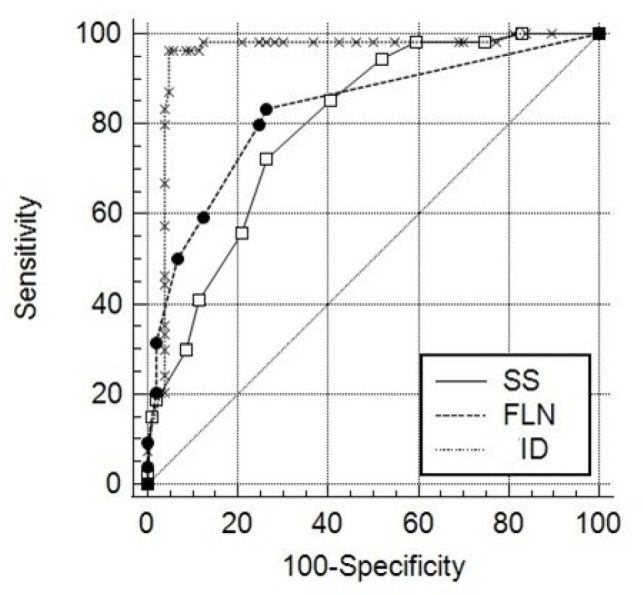
Receiver operating characteristic analysis of intrinsicoid deflection (ID), fragmented lead number (FLN), and Selvester score (SS).

AUC values for the variables analyzed by ROC curve analysis in terms of ICD ATP/shock therapy in HF patients were as follows: ID: AUC: 0.952 (95% confidence interval [CI]: 0.906 - 0.979, *P* < 0.001), FLN: AUC: 0.828 (95% CI: 0.760 - 0.883, *P* < 0.001), and Selvester score: AUC: 0.798 (95% CI: 0.727 - 0.857, *P* < 0.001). After the pairwise comparison by the Delong test, AUC of ID was statistically larger than the AUC of FLN and Selvester score (*P* = 0.002; *P* < 0.001, respectively) (Figure 2).

In the univariate logistic regression analyses, LVDD, QTc interval, FLN, ID, Selvester score, statin, and MRA predicted ICD ATP/shock therapy, while FLN, ID, and Selvester score remained independent predictors of ICD ATP/shock therapy in patients with HF (Table 3).

**Table 3:** Univariate and multivariate logistic regression analysis showing the independent predictors of shock therapy in patients with ICD.

	Univariate		Multivariate	
	Odds ratio 95% CI	*P*-value	Odds ratio 95% CI	*P*-value
LVD	1.103 (1.047 - 1.163)	< 0.001	1.107 (0.974 - 1.257)	0.119
LVH	0.643 (0.329 - 1.257)	0.197		
QRS	1.006 (0.996 - 1.015)	0.223		
Tp-e	1.006 (0.997 - 1.016)	0.207		
QTc	1.023 (1.011 - 1.035)	< 0.001	1.025 (0.997 - 1.054)	0.086
FLN	2.000 (1.599 - 2.501)	< 0.001	1.490 (1.032 - 2.151)	0.033
First intrinsicoid deflection	1.145 (1.098 - 1.194)	< 0.001	1.170 (1.091 - 1.256)	< 0.001
Selvester Score	1.508 (1.303 - 1.745)	< 0.001	2.040 (1.309 - 3.179)	0.002
Statin	0.218 (0.070 - 0.675)	0.008	0.498 (0.044 - 5.607)	0.572
MRA	0.403 (0.169 - 0.957)	0.040	0.087 (0.007 - 1.035)	0.053

CI=confidence interval; FLN=fragmented lead number; ICD=implantable cardioverter defibrillator; LVD=left ventricular diameter; LVH=left ventricular hypertrophy; MRA=mineralocorticoid receptor antagonist; Tp-e=T peak-T end

## DISCUSSION

The results of our study identified three main findings: first index, QRS interval, QTc interval, Tp-e interval, FLN, ID, Selvester score, and LVDD were higher in the ATP/shock (+) group than in the ATP/shock (-) group. Second, ID, FLN, and Selvester score were independently associated with ICD ATP/shock therapy. Third, the cutoff value of ID for ICD ATP/shock therapy was 50 msec.

There is increasing interest in ID as an electrocardiographic indicator of specific cardiac diseases when the interval > 50 msn it is called DID. The mechanism behind ID is related to the plateau phase of the myocardial action potential, which depends on calcium ion entry into cardiac myocytes via L-type calcium channels, as well as sodium ion entry through the Na/Ca exchanger. In animal models of LVH, an increase in calcium current contributes to the prolongation of the action potential through extended plateau and repolarization phases, leading to DID^[16,17]^. This dispersion during the action potential can result in re-entry phenomena at intercalated discs. Moreover, hypertrophied and ischemic human hearts have been found to have reduced gap junctions, which normally facilitate electrical current conduction between adjacent myocytes within intercalated discs. Another potential mechanism contributing to DID is increased interstitial fibrosis observed in the myocardium under failure conditions^[18]^. Due to the overlapping pathophysiological effects, both LVH and HF with reduced ejection fraction are associated with electrocardiographic findings such as QRS and QTc prolongation, which are linked to DID. Darouian et al.^[9]^ compared SCA victims with geographic controls with no SCA. They found relationship between DID > 50 msn and SCA. In our study, we analyzed patients for LVH using both echocardiographic and electrocardiographic methods. After adjusting for all these factors, ID remained an independent predictor of appropriate ICD ATP/shock therapy. Univariate analysis indicated an association between ID and ICD therapy (ATP/shock) group. After the pairwise comparison by the Delong test, AUC of ID was statistically larger than the AUC of FLN and Selvester score.

A multitude of ECG parameters associated with ventricular depolarization have been studied in relation to prolonged QRS. A prolonged QRS duration (QRSd ≥ 120 ms) is widely recognized as a determinant of risk for life-threatening arrhythmias in patients with ICDs and SCD^[19,20]^. Tp-e denotes the distance from the T-wave peak to the point of return to the isoelectric line. The Tp-e interval signifies the transmural dispersion of repolarization. While several investigations have focused on ventricular arrhythmias, there has been variability in the cutoff values for the Tp-e interval. In our study, the group receiving appropriate ICD therapy (shock/ATP) had wider QRS and longer Tp-e distance compared to the control group. This aligns with results from many studies^[21]^, which suggested that the underlying mechanism relates to the arrhythmic substrate found in ischemic cardiomyopathy or to electrical heterogeneity and depolarization/repolarization discordance in nonischemic cardiomyopathies.

Fragmented QRS (fQRS) is linked to significant scar tissue and myocardial necrosis. The presence of scar tissue and ischemic regions leads to non-homogeneous activation of the ventricular myocardium. This irregular activation can cause conduction blocks, which may result in R’ waves or notching of the S-wave, characteristics of fQRS. Areas of myocardial scar and fibrosis are typically associated with slower conduction, which is critical for the formation and maintenance of VT. The relationship between fQRS and the prediction of ICD shocks has been investigated multiple times, yielding conflicting results. These discrepancies may arise from a lack of a standardized definition for fQRS. Some studies have employed "on-off" criteria, whereby leads either show any form of fQRS or none at all. Others focus on the number of leads involved, distinguishing between one major lead or multiple contiguous leads displaying fQRS. Additionally, some studies emphasize the amplitude and width of the fQRS fragmentation. Due to these factors, the diagnostic precision of fQRS for detecting myocardial scar and its prognostic significance across various populations have demonstrated considerable variability. The morphology of fQRS is also vulnerable to both interobserver and intraobserver variability. Das et al.^[22]^ described six morphologies of fQRS, while Maheshwari et al.^[23]^ identified ten types. Although some of these morphologies are considered benign, Haukilahti et al.^[24]^ suggested that fQRS in the lateral and inferior locations may estimate an elevated risk of SCD. We looked at both the number of fragmented leads and their localization. We found the number of fragmented leads to be an independent predictor of appropriate ICD therapy (AUC: 0.828 [95% CI: 0.760 - 0.883, *P* < 0.001]). The clinical significance and the relationship between the arrhythmias and the frontal QRS-T angle is studied in many cohorts. In the study by Raposeiras-Roubin et al., 467 ischemic cardiomyopathy patients with > 90° fQRST angle found increase mortality^[25]^. Borleffs et al.^[26]^ studied 412 patients with ischemic cardiomyopathy patients and showed that a QRS-T angle exceeding 100° is a robust predictor of adequate ICD shock^[26]^; whereas according to Ozgul et al.^[27]^, patiens with fQRS-T angle > 120° received appropriate ICD shock. In these studies, mechanism was related to the arrhythmic substrate in ischemic cardiomyopathy, or electrical heterogeneity and depolarization/repolarization discordance in nonischemic cardiomyopathies but in each study, different cutoff values were correlated with clinical outcomes. In our patient cohort, no substantial difference was seen between the research groups.

In the SCD-Heft trial, there was an association between prognosis and Selvester score which is marker of myocardial scaring, derived from 12-lead ECG depending on mostly Q wave existence^[28]^. Kuyumcu et al.^[29]^ studied nonischemic cardiomyopathy patients to test Selvester score predicting appropriate ICD shock, they found the cutoff points of 5 for Selvester score to predict ICD therapy. The study by Arısoy et al.’s^[30]^ showed that the Selvester score served as an estimator for acceptable ICD shocks in both ischemic and nonischemic cardiomyopathy patients, with a cutoff value of 6.5 for its prediction.

Our study included patients with both ischemic and nonischemic cardiomyopathies, and the results were similar to those of these studies. Selvester score (*P* < 0.001) was higher in ICD therapy group. There was positive correlation between ICD shock therapy and Selvester score (*P* = 0.002, r = 0.843). ROC curve analysis showed that the cutoff value for the Selvester score to predict ICD shocks was 6.5 with a sensitivity of 72.0% and a specificity of 83% (AUC = 0.717; 95% CI: 0.627-0.807, *P* < .001).

### Limitations

There are some limitations in our study, one of these was that anatomical LVH was assessed using transthoracic echocardiography rather than more advanced and sensitive modalities, such as cardiac magnetic resonance imaging. Another limitation is that this was a single-center study, and the number of patients was small; it needs to be confirmed with larger populations. Moreover, the number of differences between the case and the control groups may lead to abnormal distribution of variables.

## CONCLUSION

Our study identified ID, FLN, and Selvester score as independent markers that can predict the need for ICD ATP/shock therapy in patients with HF. Notably, the predictive value of the ID was greater than that of the FLN and Selvester scores. ECG variables are straightforward, cost-effective, and commonly documented, providing valuable supplementary information about the benefits of ICD therapy. These markers can be used for a more comprehensive and personalized approach to patient selection for ICD therapy. By expanding beyond LVEF as the exclusive criterion for prescribing ICD therapy for primary prevention in HF, and incorporating additional predictors like ID, we may improve the selection of candidates for primary prevention ICDs.

## Data Availability

The authors declare that the data will only be available upon request to the authors.

## Artificial Intelligence Usage

The authors declare that no generative artificial intelligence or artificial ıntelligence-assisted tools were used in the preparation or writing of this manuscript.
